# The incidence of myocardial infarction and stroke in head and neck cancer patients

**DOI:** 10.1038/s41598-021-83665-4

**Published:** 2021-02-18

**Authors:** Hyun-Keun Kwon, Kyung-Do Han, Yong-Il Cheon, Sung-Chan Shin, Minhyung Lee, Eui-Suk Sung, Jin-Choon Lee, Byung-Joo Lee

**Affiliations:** 1 Department of Otorhinolaryngology-Head and Neck Surgery, College of Medicine, Pusan National University and Medical Research Institute, Pusan National University Hospital, 179 Gudeok-ro, Seo-gu, Busan, 49241 South Korea; 2 Department of Statistics and Actuarial Science, Soongsil University, Seoul, South Korea; 3 Department of Otorhinolaryngology-Head and Neck Surgery, College of Research Institute for Convergence of Biomedical Science and Technology, Pusan National University Yangsan Hospital, Yangsan, South Korea

**Keywords:** Cancer, Oncology, Risk factors

## Abstract

Various treatment modalities are used for head and neck cancer (HNC). This study analyzed the incidence and risks of myocardial infarction (MI) and stroke by cancer site and treatment modality in 22,737 patients newly diagnosed with HNC registered in the Korean National Health Insurance Service database in 2007–2013. An additional 68,211 patients without HNC, stroke, or MI were identified as the control group. The risks for MI (hazard ratio [HR] = 1.38, 95% confidence interval [CI] 1.24–1.53), stroke (HR = 1.48, 95% CI 1.37–1.60), and mortality (HR = 5.30, 95% CI 5.14–5.47) were significantly higher in the HNC group. Analysis by cancer site showed the risk of MI and mortality was highest in hypopharynx cancer, while the risk of stroke was highest in nasopharynx and paranasal sinus cancer. Analysis by treatment modality showed the highest risks for MI (HR = 1.88, 95% CI 1.31–2.69) and mortality (HR = 2.95, 95% CI 2.75–3.17) in HNC patients receiving chemotherapy (CT) alone, while HNC patients receiving CT with surgery had the highest risk for stroke (HR = 1.81, 95% CI 1.14–2.88). Careful attention to MI and stroke risks in HNC patients is suggested, especially those who received both CT and radiotherapy.

## Introduction

Head and neck cancer (HNC) is a group of cancers occurring in the tongue and oral cavity, oropharynx, nasopharynx and paranasal sinus, hypopharynx, larynx, salivary gland, and others. HNC is the ninth most common malignancy worldwide, and recent US cancer statistics indicated that more than 65,000 men and women were diagnosed with HNC in 2017^1,2^. Tobacco and alcohol are the most important risk factors for the development of HNC, especially due to their synergic effects^3^. Despite advances in various therapeutic options in the treatment of HNC, its mortality rate is high due to the high rates of local recurrence and regional metastasis^4^. Since most of these patients are diagnosed at advanced stages, they undergo multimodal therapy, thereby leading to significant acute and chronic side effects^5^.

Stroke is a common complication in cancer patients, with tumor-related disorders, coagulation disorders, infection, cancer treatment complications, and paraneoplastic causes reported as associated factors^6–11^. Radiation therapy (RT) for HNC has been shown to damage cerebrovasculature^12^; RT increases the risk of stroke by 40–46% in HNC patients^12,13^. In addition to RT, chemotherapy (CT), which is widely used in the treatment of HNC patients, causes coagulation disturbances and direct endovascular damage, leading to vascular complications^14–16^. Several studies have reported that CT increases the risk of stroke^17,18^. Both RT and CT increase the risk of thromboembolic events, which can increase the risk of myocardial infarction (MI) as well as stroke.

While many studies have reported an increased risk of stroke after treatment for HNC, none have shown an increased risk of MI. Thus, we investigated the incidence and risk of MI and stroke in HNC patients compared to those of the general population. We hypothesized that the risk of MI and stroke in HNC patients would vary between cancer site and treatment modality.

## Results

### Characteristics of the study population

The HNC and control groups had similar age and sex distributions. Of the 22,737 HNC patients, 17,166 (75.5%) were male and 5571 (24.5%) were female. The mean age in both groups was 58.65 ± 13.22 years, with most patients aged 60–79 years (10,633 of 22,737, 46.77%). The prevalence of hypertension and DM was higher in the HNC group than that in the control group (33.14% vs. 30.63% and 14.77% vs. 12.06% respectively; all *P* < 0.001) s(Table 1).

**Table 1:** Baseline characteristics of the study population.

	Control (N = 68,211)	HNC (N = 22,737)	P value
	N (%)	N (%)	
**Age (years)**			1
0–19	597 (0.88)	199 (0.88)	
20–39	4848 (7.11)	1616 (7.11)	
40–59	28,533 (41.83)	9511 (41.83)	
60–79	31,899 (46.77)	10,633 (46.77)	
≥ 80	2334 (3.42)	778 (3.42)	
Mean ± SD	58.65 ± 13.22	58.65 ± 13.22	
**Sex (male)**			1
Male	51,498 (75.5)	17,166 (75.5)	
Female	16,713 (24.5)	5571 (24.5)	
**Comorbidity**			
HTN	20,892 (30.63)	7534 (33.14)	< 0.0001*
DM	8224 (12.06)	3359 (14.77)	< 0.0001*
Dyslipidemia	9280 (13.6)	2994 (13.17)	0.095
**Income level**			< 0.0001*
Q1 (lowest)	18,962 (27.8)	6515 (28.65)	
Q2	14,599 (21.4)	4982 (21.91)	
Q3	15,826 (23.2)	5330 (23.44)	
Q4 (highest)	18,824 (27.6)	5910 (25.99)	

*HNC* head and neck cancer, *SD* standard deviation, *HTN* hypertension, *DM* diabetes mellitus.

*P < 0.05.

### MI, stroke, and mortality risks in the HNC group compared to those in the control group

**Figure 1:**
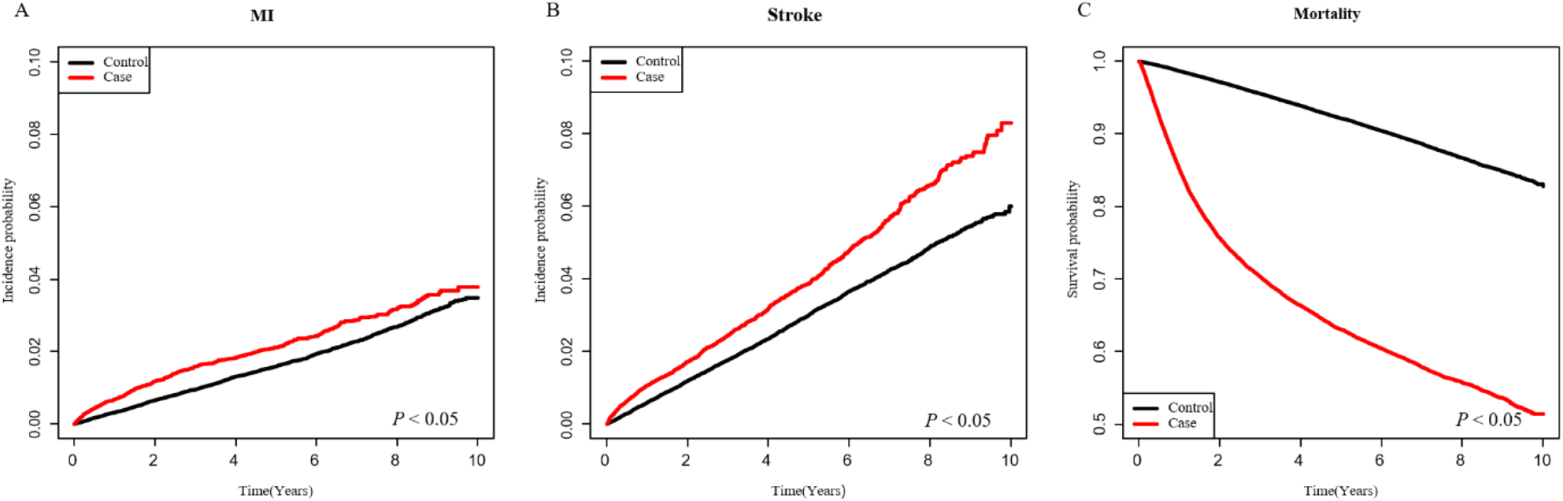
Kaplan–Meier analysis comparing incidence and survival probability between HNC patients and reference cohort. *MI *myocardial infarction.

After 5–10 years of follow-up, we identified 467 cases of MI, 887 cases of stroke, and 9206 cases of mortality in the HNC group and 1422 cases of MI, 2567 cases of stoke, and 7281 cases of mortality in the control group. Compared to the control group, the overall incidence rates of MI (4.42 vs. 3.39), stroke (8.48 vs. 6.16), and mortality (86.55 vs. 17.20) were higher in the HNC group. The risks for MI (hazard ratio [HR] = 1.38, 95% confidence interval [CI] 1.24–1.53), stroke (HR = 1.48, 95% CI 1.37–1.60), and mortality (HR = 5.30, 95% CI 5.14–5.47) were also higher in the HNC group (Table 2). The incidence probabilities of MI and stroke and the survival probabilities in the HNC and control groups are shown in Fig. 1. In the HNC group, the incidence of MI, stroke, and mortality appeared to occur immediately within 2 years, and as the follow-up period was prolonged, an increase in imbalance between groups was observed (*P* < 0.0001).

**Table 2:** Comparisons of MI, stroke, and mortality risks between the HNC and control groups.

	N	Events	IR (per 1000)	Adjusted HR (95% CI)
**MI**				
Control	68,211	1422	3.388	1 (Ref.)
HNC	22,737	467	4.424	1.376 (1.239, 1.528)*
**Stroke**				
Control	68,211	2567	6.165	1 (Ref.)
HNC	22,737	887	8.480	1.483 (1.374, 1.602)*
**Mortality**				
Control	68,211	7281	17.204	1 (Ref.)
HNC	22,737	9206	86.548	5.3 (5.138, 5.466)*

*IR* incidence rate, *HR* hazard ratio adjusted for age, sex, hypertension, diabetes mellitus, dyslipidemia, *CI* confidence interval, *HNC* head and neck cancer, *MI* myocardial infarction*.*

*P < 0.05.

### MI, stroke, and mortality risks in the HNC group by cancer site

**Figure 2:**
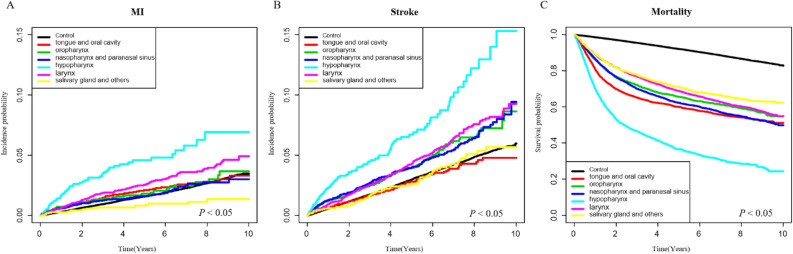
Kaplan–Meier analysis comparing incidence and survival probability by cancer site. *MI *myocardial infarction.

We investigated the risks of MI, stroke, and mortality at each HNC site. Compared to the control group, patients with hypopharynx cancer had higher risks of MI (HR = 2.40, 95% CI 1.85–3.11) and mortality (HR = 9.01, 95% CI 8.48–9.58), whereas those with nasopharynx and paranasal sinus cancer had a higher risk of stroke (HR = 2.22, 95% CI 1.91–2.58) (Table 3). The incidence probabilities of MI and stroke and survival probabilities by cancer site in the HNC and control groups are shown in Fig. 2. In the HNC group excluding the salivary gland and others group, the incidence probability of MI was similar or higher than that of the control group (*P* < 0.0001). In the HNC group excluding the tongue and oral cavity, salivary gland and others group, the incidence probability of stroke was higher than that of the control group (*P* < 0.0001). All HNC subgroups had a lower survival probability compared to the control group (*P* < 0.0001).

**Table 3:** Comparisons of MI, stroke, and mortality risks between the HNC and control groups.

Type	N	Events	IR (per 1000)	Adjusted HR (95% CI)
**MI**				
Control	68,211	1422	3.388	1 (Ref.)
Tongue and oral cavity	4319	78	4.123	1.401 (1.114, 1.762)*
Oropharynx	3143	58	4.031	1.381 (1.061, 1.797)*
Nasopharynx and paranasal sinus	4545	77	3.572	1.505 (1.195, 1.895)*
Hypopharynx	1801	59	10.184	2.396 (1.845, 3.112)*
Larynx	6462	174	5.351	1.282 (1.095, 1.502)*
Salivary gland and others	2467	21	1.697	0.696 (0.452, 1.072)
**Stroke**				
Control	68,211	2567	6.165	1 (Ref.)
Tongue and oral cavity	4319	107	5.664	1.106 (0.911, 1.343)
Oropharynx	3143	125	8.793	1.75 (1.461, 2.095)*
Nasopharynx and paranasal sinus	4545	188	8.830	2.219 (1.911, 2.577)*
Hypopharynx	1801	93	16.234	2.098 (1.705, 2.582)*
Larynx	6462	299	9.285	1.205 (1.068, 1.359)*
Salivary gland and others	2467	75	6.111	1.454 (1.155, 1.832)*
**Mortality**				
Control	68,211	7281	17.204	1 (Ref.)
Tongue and oral cavity	4319	1849	96.950	6.691 (6.354, 7.046)*
Oropharynx	3143	1160	80.011	5.389 (5.064, 5.736)*
Nasopharynx and paranasal sinus	4545	1895	87.421	7.395 (7.025, 7.784)*
Hypopharynx	1801	1208	205.639	9.014 (8.477, 9.584)*
Larynx	6462	2309	70.304	3.312 (3.16, 3.472)*
Salivary gland and others	2467	785	63.280	5.26 (4.883, 5.666)*

*IR* incidence rate, *HR* hazard ratio adjusted for age, sex, hypertension, diabetes mellitus, dyslipidemia, *CI* confidence interval, *MI* myocardial infarction.

*P < 0.05.

### MI, stroke, and mortality risks in the HNC group by age and sex

The risks of MI, stroke, and mortality were analyzed according to age and sex within the HNC group. When analyzed by age, the risks for MI (HR = 8.34, 95% CI 4.18–16.63), stroke (HR = 10.99, 95% CI 6.04–19.97), and mortality (HR = 8.43, 95% CI 7.38–9.63) were highest in people over 80 years of age. When analyzed by sex, the incidence rates and risks of MI, stroke, and mortality were higher in male patients (Table 4).

**Table 4:** MI, stroke, and mortality risks in the HNC group by age and sex.

Type	N	Events	IR (per 1000)	Adjusted HR (95% CI)
**MI**				
Age				
0–19	199	1	0.836	0.631 (0.082, 4.851)
20–39	1616	12	1.345	1 (Ref.)
40–59	9511	121	2.485	1.543 (0.85, 2.801)
60–79	10,633	305	6.814	3.872 (2.155, 6.958)*
≥ 80	778	28	14.106	8.341 (4.183, 16.632)*
Sex				
Male	17,166	388	4.987	1 (Ref.)
Female	5571	79	2.847	0.646 (0.506, 0.824)*
**Stroke**				
Age				
0–19	199	2	1.660	0.965 (0.221, 4.222)
20–39	1616	15	1.683	1 (Ref.)
40–59	9511	244	5.041	2.671 (1.583, 4.506)*
60–79	10,633	584	13.232	6.558 (3.908, 11.005)*
≥ 80	778	42	21.631	10.987 (6.044, 19.973)*
Sex				
Male	17,166	749	9.728	1 (Ref.)
Female	5571	138	4.998	0.568 (0.473, 0.682)*
**Mortality**				
Age				
0–19	199	33	27.380	0.657 (0.46, 0.938)*
20–39	1616	363	40.587	1 (Ref.)
40–59	9511	2864	58.532	1.396 (1.251, 1.558)*
60–79	10,633	5330	117.720	2.964 (2.66, 3.302)*
≥ 80	778	616	306.089	8.43 (7.379, 9.632)*
Sex				
Male	17,166	7339	93.527	1 (Ref.)
Female	5571	1867	66.919	0.879 (0.835, 0.925)*

*IR* incidence rate, *HR* hazard ratio adjusted for age, sex, hypertension, diabetes mellitus, dyslipidemia, *CI* confidence interval, *MI* myocardial infarction.

*P < 0.05*.*

### MI, stroke, and mortality risks in the HNC group by treatment modality

The risks of MI, stroke, and mortality were analyzed according to treatment modality in the HNC group. Compared to patients with no treatment, the risk of MI was highest in patients receiving CT alone (HR = 1.88, 95% CI 1.31–2.69), the risk of stroke was highest in patients receiving both surgery and CT (HR = 1.81, 95% CI 1.14–2.88), and the risk of mortality was highest in patients receiving CT alone (HR = 2.95, 95% CI 2.75–3.17) (Table 5).

**Table 5:** MI, stroke, and mortality risk in the HNC group by treatment modality.

	N	Events	IR (per 1000)	Adjusted HR (95% CI)
**MI**				
No treatment	7056	151	4.15758	1 (Ref.)
Surgery alone	2630	51	3.45079	0.845 (0.615, 1.161)
CT alone	1783	38	5.82242	1.876 (1.308, 2.689)*
RT alone	3567	77	4.582	1.032 (0.783, 1.359)
Surgery + CT	330	4	2.99694	0.854 (0.316, 2.309)
Surgery + RT	1779	29	3.17693	0.836 (0.562, 1.245)
CT + RT	4071	92	6.01654	1.576 (1.209, 2.055)*
Surgery + CT + RT	1521	25	4.65853	1.212 (0.79, 1.859)
**Stroke**				
No treatment	7056	339	9.4626	1 (Ref.)
Surgery alone	2630	97	6.6152	0.719 (0.574, 0.902)*
CT alone	1783	47	7.2256	1.021 (0.751, 1.389)
RT alone	3567	133	7.959	0.805 (0.659, 0.985)*
Surgery + CT	330	19	14.4549	1.81 (1.139, 2.877)*
Surgery + RT	1779	61	6.7278	0.787 (0.599, 1.034)
CT + RT	4071	140	9.2268	1.121 (0.917, 1.371)
Surgery + CT + RT	1521	51	9.5414	1.17 (0.869, 1.576)
**Mortality**				
No treatment	7056	2604	71.135	1 (Ref.)
Surgery alone	2630	552	37.116	0.535 (0.488, 0.586)*
CT alone	1783	1144	174.063	2.954 (2.752, 3.17)*
RT alone	3567	1272	74.98	0.974 (0.911, 1.042)
Surgery + CT	330	194	145.275	2.257 (1.95, 2.612)*
Surgery + RT	1779	571	62.182	0.913 (0.833, 0.999)
CT + RT	4071	2004	129.861	1.849 (1.742, 1.962)*
Surgery + CT + RT	1521	865	160.094	2.282 (2.11, 2.468)*

*IR* incidence rate, *HR* hazard ratio adjusted for age, sex, hypertension, diabetes mellitus, dyslipidemia, *CI* confidence interval, *MI* myocardial infarction, *RT* radiation therapy, *CT* chemotherapy.

*P < 0.05.

## Discussion

To our knowledge, this is the first nationwide study to investigate the relative risks of MI, stroke, and mortality incidences among HNC patients who underwent surgery, CT, and RT compared to a general non-cancer control population matched for age and sex. After adjusting for age, sex, and comorbidities such as hypertension (HTN), diabetes mellitus (DM), and dyslipidemia, the risks of MI, stroke, and mortality were significantly higher in the HNC group compared to those in the control group. Analysis by cancer site showed the highest risk of MI and mortality in patients with hypopharynx cancer, while the risk of stroke was highest in patients with nasopharynx and paranasal sinus cancer. Analysis by treatment modality showed high incidences of MI, stroke, and mortality for treatment with CT alone or with surgery or RT. After adjusting for age, sex, and comorbidities, the risks of MI and stroke were highest in patients who received CT alone or with surgery.

HTN, DM, and dyslipidemia are well-known risk factors for vascular events such as stroke and MI^19,20^. In addition, studies on the effects of HTN, DM, and dyslipidemia on the development of cancer are ongoing. A previous large, population-based case–control study reported inverse associations between type II DM, HTN, dyslipidemia, and HNC^21^. However, in the present study, the incidence of HTN and DM were higher in HNC patients than in the controls. Even after adjusting for the risk factors of vascular events such as DM, HTN, and dyslipidemia, the risks of stroke and MI remained higher in HNC patients.

Previous studies reported the highest and lowest 5-year HNC survival rates for lip cancer and hypopharyngeal cancer, respectively^22,23^. The 8th edition of the American Joint Committee on Cancer (AJCC) divides lip cancer into mucosal and cutaneous lip. Mucosal lip is included in the oral cavity while cutaneous lip is included in cutaneous carcinoma of the head and neck^24^. Thus, this study included mucosal lip cancer as an oral cavity cancer. When lip cancer was excluded from previous studies, salivary gland cancer had the highest 5-year survival rate in HNC^22,23^. In the present study, larynx cancer showed the lowest mortality risk and hypopharynx cancer showed the highest mortality risk in HNC patients.

Several HNC cohort studies have investigated the risk of stroke following treatment. A population-based study similar to the present study reported that HNC patients are at increased risk of developing stroke, especially the young age group and those who received both RT and CT^25^. In our study, HNC increased the risk of stroke by 48.3% compared to control. However, unlike the previous study, the stroke risk increased with age and the stroke risk was highest in patients in the present study who received both surgery and CT.

Many studies have shown that RT is associated with increased risks of cerebrovascular disease in HNC patients. The exact mechanism of radiation-induced carotid or cerebral artery injury is not clear but is thought to be due to endothelial dysfunction, injury and occlusion of the vasa vasorum, and accelerated atherosclerosis^26,27^. Several population-based cohort studies reported increased cerebrovascular risk in HNC patients who underwent RT alone compared to that in patients who underwent surgery with or without radiation^12,28^. A recent population-based cohort study reported that the risk of stroke increased with RT after adjusting for other socioeconomic and clinical risk factors. The authors suggested that attention should be paid to advanced-age patients with low socioeconomic status and known clinical risk factors of stroke^13^. However, in the present study, patients who received RT alone or surgery with or without radiation had lower risks of stroke than those who received no treatment and the stroke risk was highest in patients who underwent both surgery and CT.

The risk of thromboembolic events increases after chemotherapy in patients with various cancers. Ischemic cerebrovascular accidents caused by chemotherapy have been reported in various cancers, including HNC, breast cancer, and lymphoma^17,29–31^. The mechanism of chemotherapy-induced thromboembolism is also not clear, although tumor embolization, vasculitis, nonbacterial thrombotic endocarditis, consumption coagulopathy, or complications related to chemotherapeutic agents have been suggested as causes of thromboembolism^18,32,33^. Cisplatin is the main anti-cancer drug in CT of HNC and commonly causes cerebrovascular events^18^. After CT with cisplatin, cerebrovascular accidents occur due to vasospasm, hyperreninemia or hyperaldosteronemia, platelet hyperaggregation, decreased tissue activators, endothelial dysfunction, and elevated serum cholesterol levels^18,34–37^.

There have also been reports of chemotherapy-induced cardiotoxicity (CIC). CIC occurs due to the direct effect of the drug on the cardiovascular system or indirect effects due to thrombogenic status or hemodynamic flow alterations^38^. The risk of CIC development is affected by several factors, including the type of chemotherapy agent, the administered dose, and the drug administration rate^39,40^. However, to our knowledge, there have been no reports of increased MI after treatment in HNC patients. Although the mechanism is not clear, the results of this study showed an increased incidence of MI after treatment in HNC patients, especially in those who received CT alone.

Contrary to previous studies, where the risk of stroke increases due to RT, the group receiving both surgery and CT in the present study showed the highest stroke incidence rate or HR. According to the results of this study, CT appeared to increase the risks of MI, stroke, and mortality more than RT. Patients who received CT alone are likely to have received palliative CT due to advanced stage or underlying diseases. Therefore, the highest mortality rate among patients who received CT alone may have been affected by complications such as MI and stroke.

This study has several limitations. First, alcohol consumption, smoking, and body mass index (BMI) are important risk factors for stroke and MI as well as HNC. However, the KNHIS data did not provide information on alcohol consumption, smoking and BMI. Second, the type and administered dose of CT agent and the intensity of RT used in treatment could not be confirmed. Finally, our study was based on an Asian population; thus, the results may be different in Western populations. Therefore, our results need to be analyzed in other ethnic groups.

In conclusion, the results of our study showed increased MI and stroke risks among HNC patients compared to those in the general non-cancer population. After adjusting for age, sex, and comorbidities, the risks of MI and mortality were highest in hypopharynx cancer, while the risk of stroke was highest in nasopharynx and paranasal sinus cancer. Analysis by treatment modality showed that the risks of MI, stroke, and mortality were highest in the group that received CT alone or with surgery. Therefore, careful attention to MI and stroke risk for HNC patients is suggested, especially in those who receive both CT and RT.

## Methods

### Data source

The Korean National Health Insurance Service (KNHIS) is the public medical insurance system administered by the Ministry for Health, Welfare and Family Affairs^41^. As a compulsory social insurance system, the KNHIS program includes approximately 97% of the entire Korean population. Individuals in the lowest income level bracket (approximately 3% of the population) are covered by the Medical Aid program. The KNHIS database includes patient demographics and records on diagnosis (based on International Classification of Diseases, 10th revision, Clinical Modification [ICD-10-CM] codes), interventions, and prescriptions^42^. Additionally, the KNHIS provides a biennial national health check-up program for all beneficiaries aged ≥ 40 years old or employees regardless of age^43^. This program aims to detect cardiovascular risk factors, including age, sex, smoking status, alcohol consumption, hypertension, DM, and dyslipidemia, for subsequent educational counseling.

The KNHIS database has been widely used in numerous epidemiological and health services-related research studies^44^. Its detailed profile and configuration have been previously described^45,46^. The research protocol was approved by the Institutional Review Board of the Pusan National University Hospital and informed consent was waived.

### Study population and design

From KNHIS data, we identified 22,737 cases of HNC (ICD-10-CM code C01–14, C30–33, C43.2, and C44.2), newly diagnosed in 2007–2013. Patients with prior stroke (ICD-10-CM code I63–64) or MI (ICD-10-CM code I21–22) were excluded. HNC included malignant neoplasms of the tongue and oral cavity (19.0%), oropharynx (13.8%), nasopharynx and paranasal sinus (20.0%), hypopharynx (7.9%), larynx (28.4%), and salivary gland and others (10.9%). The follow-up period was calculated from the date of HNC diagnosis. For the selection of the control group, we used a ratio of 1:3; thus, 68,211 individuals free from any cancer and prior stroke or MI and matched for age, sex. Baseline comorbidities were evaluated during the study period. Baseline characteristics of the comorbidities were extracted from the medical claims according to the ICD-10-CM codes. We included hypertension, DM, and dyslipidemia. Income level was categorized into quartiles based on individual insurance contribution; the medical aid population (~ 3% of Korean population) were merged with the lowest income quartile group for the analyses. The index dates were randomly selected as the control group.

The design of this study was as follows; (1) analyze the incidence and risks of MI, stroke, and mortality in the total HNC and control groups; (2) analyze the incidence and risk of MI, stroke, and mortality by HNC site (tongue and oral cavity, oropharynx, nasopharynx and paranasal sinus, hypopharynx, larynx, salivary gland, and others); and (3) analyze the incidence and risks of MI, stroke, and mortality by age, sex, and treatment modality (no treatment, surgery alone, RT alone, CT alone, surgery and CT, surgery and RT, concurrent chemoradiation therapy (CCRT), surgery and CCRT) in the HNC group.

### Statistical analysis

We compared the distributions of demographic status and comorbidity between the HNC and control groups using chi-square tests for categorical variables and *t*-test for continuous variables. We calculated the incidence rates of MI, stroke, and mortality by dividing the number of incident cases by the total follow-up period. The incidence rates of MI, stroke, and mortality are presented as 1000 person-years. The hazard ratios (HRs) and 95% confidence intervals (95% CIs) describing the risks of stroke, MI and mortality were calculated using Cox regression models adjusted for age, sex, and other comorbidities, including hypertension, DM, dyslipidemia, and income. The incidence and survival probability was calculated by using the Kaplan–Meier curves, and the log-rank test was performed to analyze differences among the groups.

The HNC group was compared to the control group. To assess the risk of MI, stroke, and mortality by HNC site, the study population was divided into six subgroups based on ICD-10-CM code (tongue and oral cavity, oropharynx, nasopharynx and paranasal sinus, hypopharynx, larynx, salivary gland, and others). To assess the effect of therapy, the study population was divided into seven subgroups based on treatment modality (no treatment, surgery alone, RT alone, CT alone, surgery and CT, surgery and RT, concurrent chemoradiation therapy (CCRT), surgery and CCRT). Statistical analyses were performed using SAS version 9.2 (SAS Institute, Cary, NC, USA). A two-sided *P-*value < 0.05 was considered to indicate statistical significance.

## Data Availability

The datasets generated during and/or analyzed during the current study are available from the corresponding author on reasonable request.
